# Bariatric Surgery and Metabolic Bone Disease: Crosstalk between Muscle, Adipose Tissue, and Bone

**DOI:** 10.1007/s11695-026-08488-6

**Published:** 2026-02-24

**Authors:** Leandro Borges, Andréa Bezerra, Giorjines Boppre, Elaine Hatanaka, Hélder Fonseca

**Affiliations:** 1 Research Center in Physical Activity, Health and Leisure (CIAFEL), Faculty of Sport, University of Porto, Porto, Portugal; 2 Instituto de Ciências da Atividade Física e Esportes (ICAFE), Universidade Cruzeiro do Sul, São Paulo, Brazil; 3 Laboratory for Integrative and Translational Research in Population Health (ITR), Porto, Portugal; 4 Nucleus of Research in Human Movement Science, Universidad Adventista de Chile, Chillán, Chile

**Keywords:** Obesity, Weight loss, Biochemical markers of bone turnover, Exercise

## Abstract

After bariatric surgery (BS), there is an increased risk of bone health issues due to restricted nutrient intake, malabsorption, insufficient supplement adherence, and reduced mechanical loading from significant weight loss. We aimed to provide a review of the current understanding of post-BS human physiology and the relevant animal models employed to investigate bone metabolism. Muscle-derived signals influence bone metabolism through mechanisms beyond just mechanical forces, highlighting the relevance of skeletal muscle as an endocrine organ to regulate bone health. BS patients often have high parathyroid hormone (PTH) due to calcium and vitamin D deficiency, which inhibits bone formation. Bone, muscle, and fat tissues communicate via cytokines, and myostatin negatively affects bone by increasing sclerostin, Dickkopf-1, and nuclear factor kappa β ligand, leading to higher bone resorption. Moreover, BS leads to changes in gastrointestinal signaling and gut peptides like glucagon-like peptides (GLP)-1 and peptide YY (PYY). Additionally, exercise may impact bone hormonal signaling, potentially lowering sclerostin and linking mechanical loading to osteoporosis. Nevertheless, there remains limited evidence regarding the association between bone remodeling and changes in these proteins post-BS. As outlined, future studies are required to validate findings from preclinical models and to adequately test these hypotheses in humans, especially regarding a better understanding of cytokines, ghrelin, sclerostin, PTH, cholecystokinin, GLP-1, and PYY physiology, their roles in obesity and BS, and their therapeutic potentials.

## Introduction

Bariatric surgery (BS) has emerged as an effective long-term treatment strategy for patients with severe obesity [1]. However, BS causes anatomical changes in the gastrointestinal (GI) tract, impacting its normal function and nutrient flow [2]. Due to their high reoperation rates, limited long-term effectiveness, and significant side effects, procedures such as adjustable gastric banding, jejunoileal bypass, and vertical banded gastroplasty are being considered less often. However, sleeve gastrectomy (SG) and Roux-en-Y gastric bypass (RYGB) (53.6% and 30.1% of all BS, respectively) are the two most commonly performed procedures worldwide, with many reported benefits [3]. The SG has become the most common bariatric procedure due to being an easier technique, with shorter operation time and similar short-term weight loss (WL) and clinical outcomes compared with RYGB. It involves transection along the greater gastric curvature, creating a tube-like new stomach, and removing the fundus and body [4]. In the RYGB, the stomach is divided, producing a small gastric pouch (20–30 mL), anastomosed with the mid-jejunum to form the Roux or alimentary limb. In this procedure, bile acids and pancreatic secretions drainage are made possible by the anastomosis of the biliopancreatic limb with the jejunum [3].

The efficacy of BS is not only associated with WL but also with the decrease or even resolution of many associated comorbidities [5]. Clinical evidence shows that surgeries with manipulations of the GI tract may result in the remission of type 2 diabetes mellitus (T2DM) due to its effects on metabolic regulation [6]. Moreover, a longitudinal cohort study by Gero et al. evaluated patients with severe obesity who underwent RYGB over five years [7]. They reported a significant improvement in the lipid profile six months after surgery and observed that this improvement was sustained for at least five years. The changes in lipid profile found in this study also resulted in a significant reduction in cardiovascular risk (27%) after the first postoperative year.

Surgical procedures with intestinal bypass, excluding the duodenum and jejunum, have been shown to exert beneficial effects in addition to those mediated by WL alone [8, 9]. However, ongoing concerns revolve around possible long-term adverse effects on bone health following BS. Patients with obesity undergoing BS often present an increased risk of bone-related problems attributed to limited nutrient intake, malabsorption, and insufficient compliance with recommended supplements.

This review aims to discuss the present knowledge regarding human physiology after BS, along with the animal models used to study bone metabolism. Additionally, we explored the various functions of bone-derived signaling and delved into the complex interactions between muscle, adipose tissue, and bone.

## Bariatric Surgery

BS has emerged in the last decade as the most effective long-term treatment strategy for patients with severe obesity (BMI ≥ 40–35 kg/m^2^ with associated complications) [1]. Moreover, bariatric procedures change the GI tract anatomy, affecting its function, namely the flow of nutrients and hormone secretion [2]. Some surgery techniques, like adjustable gastric banding, jejunoileal bypass, and vertical banded gastroplasty, have fallen out of favor due to their significant adverse effects, frequent need for reoperation, and limited long-term success. Conversely, SG and RYGB now dominate the field, making up 55.4% and 39.6% of the most performed bariatric procedures globally, respectively. This popularity can be ascribed to the many documented benefits of these techniques [3].

SG is a more straightforward technique, requires a shorter operation time, and has similar WL and metabolic outcomes compared with RYGB [4]. It involves transection along the greater stomach curvature and removal of the fundus and body, creating a tube-like new gastric structure (4). In the RYGB, the stomach is divided, producing a small proximal gastric pouch with 20-30 mL, which is anastomosed with the mid-jejunum to form the Roux or alimentary limb [5, 7]. In this procedure, bile and pancreatic secretions drainage are made possible by the anastomosis of the biliopancreatic limb with the jejunum [3]. Due to the high reoperation rates presented in these surgeries, limited long-term effectiveness, and significant side effects, procedures such as adjustable gastric banding, jejunoileal bypass, and vertical banded gastroplasty have mostly been abandoned in the present.

The efficacy of BS is also associated with the decrease or even resolution of comorbidities related to long-term severe obesity [5]. A longitudinal cohort study by Gero et al. evaluated 1048 patients with severe obesity who underwent RYGB over five years. They reported an improvement in the lipid profile six months after surgery and observed that this improvement was sustained for at least five years [7]. These changes in the lipid profile found in this study (reduction of total and LDL cholesterol, and triglyceride levels, as well as an increase in HDL levels) also resulted in a significant decrease in cardiovascular risk (27%) after the first postoperative year. BS has also been considered a crucial approach in treating individuals living with severe obesity and T2DM.

In a systematic review and meta-analysis [8] of randomized clinical trials comparing surgical with non-surgical treatment of obesity, BS was associated with greater WL, a higher rate of T2DM and metabolic syndrome remission, lipid profile, and quality of life improvement, as well as substantial reductions in the need for medication. Additionally, BS significantly improves non-metabolic conditions such as obstructive sleep apnea syndrome, leading to resolution or improvements in a large percentage of cases [9].

### Bariatric Surgery and Bone Loss

Bone is a dynamic organ with paracrine and endocrine functions that are constantly remodeled [10]. Bone turnover is modulated by the coupling of bone formation and resorption [11], which allows the renewal of the bone matrix during the remodeling process. Osteoblasts and osteoclasts are the primary cells responsible for the remodeling process through synthesizing and secreting proteins at the bone surface, which culminates in extracellular matrix (ECM) degradation, performed by osteoclasts and subsequent ECM formation through osteoblasts action [12]. Osteocytes, which regulate the activity of these cells, are former differentiated osteoblasts surrounded by ECM. Osteocytes are recognized as the most sensitive cells to mechanical stimulation, thereby fundamental to bone structural adaptative responses to loading [13]. This daily process of synthesis and secretion of a great number of proteins involved in the remodeling process needed to maintain the integrity of the skeleton is energetically expensive to the body [14]. Beyond their paracrine function, bone cells, such as osteoblasts, can also interact with other systemic hormones, such as insulin secreted from pancreatic islets and leptin secreted from adipocytes, thereby evidencing that bone influences the whole body’s energetic homeostasis [15].

Although BS results in significant and long-term WL in most patients and the improvement or resolution of several obesity-related comorbidities, there is some concern about the potential long-term negative consequences of BS, particularly in terms of the development of metabolic bone disease and increased risk of fracture [16]. Evidence has indicated that BS may negatively affect bone health [17]. Longitudinal data show that during the first year after BS, a significant bone mass (BM) loss occurs, and this decrease may continue even after the stabilization of the WL. Additionally, the reduction in BM after surgery is accompanied by a sharp increase in biochemical markers of bone remodeling [18], showing that BS substantially increases bone turnover, favoring bone resorption.

A prospective study [19] assessed areal bone mineral density (aBMD) by dual-energy X-ray absorptiometry (DXA) and volumetric bone mineral density (vBMD) by quantitative computed tomography. The study included 48 adults with obesity (27 premenopausal women, 11 postmenopausal women, and 10 men) before, six months, and 12 months after RYGB. It was estimated that aBMD of the femoral neck decreased by 5% and 8% between 6 and 12 months after surgery. Additionally, vBMD of the lumbar spine decreased on average by 6.6% and 8.1% at 6 and 12 months after surgery. Notoriously, the decrease at 12 months was higher in post-menopausal (11.6%) compared to pre-menopausal women (6.0%). Cortical porosity also increased after BS in the radio and tibia, and these increases were again higher in post-menopausal (51.4%) compared to pre-menopausal women (18.3%) or men (3.0%). The results of this study suggest that RYGB leads not only to a decrease in BM, both in the axial and appendicular skeleton, affecting both the trabecular and cortical compartments, but also that these changes are more evident in post-menopausal women, who are, from the outset, the population at higher risk of developing osteoporosis and fragility fractures.

A longitudinal study [20] followed 25 patients with obesity for two years, with assessments before BS and again at 12 and 24 months of follow-up after surgery. The aBMD of the hip and lumbar spine was assessed by DXA, while vBMD and trabecular microarchitecture were evaluated at the distal radius and tibia by high-resolution peripheral CT (HR-pQCT). The study found that, despite WL stabilized between 12 and 24 months after BS, a decrease in vBMD and a deterioration of the trabecular microarchitecture continued throughout the 24 months of follow-up. DXA assessments showed a significant decrease in aBMD compared to baseline in the total hip (-8.2%) and lumbar spine (-3.5%) BMD at 12 months, and of about − 10.5% and − 5.3% at 24 months, respectively. The total vBMD of the radius (-4.3%) and tibia (-7.2%) also decreased during the study period [20]. Assessing the changes in BMD and trabecular microarchitecture in patients with severe obesity over the first 12 months after RYGB, Frederiksen et al. also reported that there was a noticeable decline in both cortical and trabecular vBMD and microarchitecture [21]. Specifically, there was a 5% reduction in estimated bone strength at the radius and a 7% reduction at the tibia, both statistically significant. The deterioration observed in the first year was either matched or surpassed by declines in the subsequent 12 months. Notably, although there was a significant increase in bone turnover markers and a decrease in leptin and insulin levels at 24 months, these changes were most prominent at the end of the first year and remained relatively stable from months 12 to 24. The mechanisms generally proposed for the increase in bone resorption and decrease in BM after BS include a lower mechanical load on bone structures, changes in hormone levels with effects on bone metabolism, and nutrient malabsorption, especially calcium and vitamin D [22]. An increase in bone resorption and a consequent decrease in BMD after BS contribute to a reduction in bone strength, which may predispose these patients to an increased risk of fractures [23]. Given the mechanosensitivity of bone tissue, the substantial WL experienced after surgery promotes a reduction in the mechanical loading experienced by bone tissue, and this effect probably plays a role in bone loss after BS [24]. On the other hand, a randomized controlled trial [25] has demonstrated that the link between weight and bone loss appears to stem from other physiological factors, such as the loss of fat and lean mass (LM), rather than a decrease in gravitational loading per se. Therefore, it is predictable that the marked WL occurring after BS leads to a reduction of mechanical stimulation on bone tissue and a consequent increase in the serum concentration of sclerostin secreted by osteocytes, thereby decreasing bone formation. A WL of about 10% brought by diet in patients with obesity was associated with an approximately 10% increase in sclerostin concentration [26]. In addition, this relationship may also be influenced by the role of sclerostin on lipid and glucose metabolism. Current research highlights the pivotal role of sclerostin in regulating these metabolic processes, particularly in individuals with osteoporosis, obesity, and diabetes [27]. Similarly, in patients with obesity submitted to BS, there is a significant and sustained increase in sclerostin concentration, and this increase is correlated with the increase in C-terminal telopeptide, a marker of bone resorption [28].

It is known that the overweight or obese is generally associated with higher LM [29]. Also, the dynamic loads imposed by skeletal muscle contraction are more anabolic to bone compared to the static loads resulting from the accumulation of excess fat mass (FM) [30]. Thus, because the majority of patients undergoing BS lose a high percentage of LM after surgery [31], BM loss may also be related to the reduction of mechanical stimulation induced by skeletal muscle contraction.

BS alters the secretion of hormones from adipose tissue and the GI tract, which, in turn, also affects bone metabolism [32], Vitamin D and calcium deficiency are common adverse effects after BS [33]. Changes induced by surgical procedures in the anatomy of the small intestine, especially after RYGB, lead to malabsorption of these nutrients, since the duodenum and proximal jejunum are the main sites for calcium absorption, while vitamin D is preferably absorbed in the jejunum and ileum. Vitamin D deficiency persists in the long-term, both after malabsorptive and restrictive procedures, and the reduction in calcium absorption can also occur after procedures in which there is no change in the anatomy of the intestine due to the reduction of gastric acidity and gastric volume [22]. The decrease in calcium and vitamin D absorption after BS leads to a compensatory hormonal response mediated by parathyroid hormone (PTH) to keep calcaemia within physiological limits by increasing calcium mobilization from its largest reservoir, which is bone tissue, gradually favoring the loss of BM [34]. Patients undergoing BS often exhibit high levels of PTH and secondary hyperparathyroidism due to calcium and vitamin D deficiency [35].

## Bone as an Endocrine Organ

Bone-derived secreted factors, such as fibroblast growth factor (FGF) 23 [36] and osteocalcin (OCN) [37], seem to trigger different effects in other body physiological processes that are not directly involved with bone tissue metabolism. Bone-derived FGF23, for instance, is related to whole-body phosphate homeostasis, acting in kidney phosphate reabsorption inhibition and 1,25- dihydroxy vitamin D3[1,25(OH)2D3] biosynthesis suppression [38], as can be observed by 1,25-dihydroxy vitamin D decreases and hypophosphatemia after FGF23 administration [39] and by an enhanced renal phosphate reabsorption and high serum 1,25(OH)_2_D in mutant mice lacking FGF23 [40].

OCN, in turn, can be found in the circulation in two forms, γ-carboxylated (GlaOCN) and uncarboxylated (GluOCN), but only the latter has demonstrably a hormonal function [41]. Experimental in vivo and in vitro studies reported that OCN deficiency led to abdominal fat accumulation [15], male fertility alterations, such as lower testosterone circulating levels [42], and even affects brain development [43]. OCN deletion in mice resulted in lower muscle performance during exercise [44] and higher muscle atrophy in rats submitted to hindlimb suspension [45]. In humans, circulating OCN levels are inversely correlated with insulin resistance [46], fasting insulin, fasting glucose, BMI, and body fat [47]. Similarly, patients submitted to biliopancreatic diversion (BPD) presented an increase in GluOCN levels at three- and 12-months post-surgery, which negatively correlated with fasting insulin and HOMA-IR, and positively correlated with insulin sensitivity, suggesting a possible glucose homeostasis regulation by bone remodeling after surgery [48]. Moreover, as a bone formation biomarker, OCN was found to decrease in patients with T2DM submitted to RYGB, which was followed by lumbar spine and total hip BMD losses, evidencing a decrease in the surgical group [49]. Interestingly, the increases in GluOCN, which seem to improve insulin metabolism, result from previously increased bone resorption, remaining the controversial observation that the negative effect of BS in BM may be the trigger for the improvement in systemic insulin sensitivity through GluOCN increase [48]. Therefore, it seems that OCN, more specifically the GluOCN, can regulate whole-body energy metabolism by improving insulin sensitivity in the liver and adipose tissue, insulin production by the pancreas, as well as energy expenditure in skeletal muscle [10].

Insulin might interact directly with the skeletal system due to the presence of insulin growth factor 1 (IGF-1) receptors in osteoblasts, and indirectly due to the regulation of blood glucose levels, as well as PTH, IGF-1, and vitamin D levels [50]. Insulin may also affect BMD through increases in sex hormones and saturated fatty acid availability [51]. The role of insulin, promoting osteoblast proliferation and increasing bone remodeling, culminates in GluOCN increase [52], whereas insulin resistance decreases OCN expression during osteogenic differentiation [53]. As previously mentioned, GluOCN also regulates insulin secretion and sensitivity in peripheral tissues, evidencing a bone-pancreas endocrine loop [53].

Of note, some adipokines also seem to be responsive to insulin plasma concentrations. For instance, lower adiponectin expression is correlated with higher insulin levels [54], whereas leptin seems to directly correlate with insulin resistance [55] and might indirectly affect bone health. A recent meta-analysis also evidenced reduced insulin resistance after BS [56]. Due to the anabolic effect of insulin in bone, the decrease in insulin levels after BS is speculated to be related to the observed decreases in BMD. However, further studies are necessary to elucidate the effect of insulin on bone health after BS.

## A Muscle-adipose Tissue-bone Crosstalk

### Muscle-adipose-bone Crosstalk and Bariatric Surgery: Possible Implications

Among the many interactions between bone tissue and other body systems, there is an interesting crosstalk between bone, fat, and skeletal muscle, which raises a particular concern regarding BS-induced body composition changes and possible musculoskeletal issues that can be developed in the long term by this population.

Skeletal muscle and bone can interact not only through direct mechanical connection but also through paracrine signals [14] performed by myokines and osteokines, as well as growth-like factors, which affect, afterward, musculoskeletal metabolism [11]. Growth factors, such as IGF-1 [57] and FGF-2 [58], are documented to increase osteoblast proliferation and accelerate bone formation [59]. Interestingly, despite IGF-1 being correlated with a lower inflammatory pattern in patients with obesity and with positive effects in BMD [60], some studies evidence reduced levels of IGF-1 at three [61] and six [62] months after SG, even with an improved chronic low-grade inflammation. However, at 12 months post-RYGB, a study reported increased IGF-1 levels in bariatric patients (*n* = 116) [63], showing that maybe longer follow-ups are necessary to promote a significant decrease in whole-body systemic inflammation and, thereby, observe the IGF-1 response to this surgical intervention.

Irisin is another hormone involved in skeletal muscle-bone crosstalk that is correlated with bone health through an increase in osteoblastogenesis and bone formation [64]. Weekly low doses of r-irisin (100 µgkg^− 1^) in young mice were able to stimulate increases in tibia cortical BM and strength [64], highlighting that irisin can interact with bone in an anabolic way. Beyond being released during muscle contraction, recent evidence has shown that irisin can also be considered an adipokine since it is secreted by human visceral and subcutaneous adipose tissue, which could justify higher irisin levels in individuals with obesity [65]. Regarding irisin response after BS, the literature is still divergent. Some evidence decreases in irisin levels at six and 12 months after RYGB and SG [66], whereas others report an increase post-SG [67], or laparoscopic adjustable gastric banding (LAGB) [68], or even no changes [69] at six months after RYGB. An interesting pilot study observed a bidirectional response in irisin levels in the first month after RYGB related to the patients (*n* = 12) baseline values [70]. The results suggest that patients with lower baseline irisin concentration increased their levels, whereas those with higher irisin values decreased in the first months after BS. This response might be explained by the energy deprivation in the first month post-BS, which promotes a negative nitrogen balance and, consequently, can compromise muscle physiology and metabolism. This hypothesis could also explain the recovery in irisin levels nine months post-BS in this same study [70]. A divergent irisin response was also verified in adolescents after BS, suggesting a potential heterogeneity in response to RYGB. Notwithstanding, patients with greater changes in irisin were associated with higher BMI losses [67]. Despite finding interesting patterns, these studies have a small sample size as a limitation, making the effective response of irisin to BS unclear until the moment.

The effect of the interaction of some myokines with bone tissue is still controversial. For instance, myostatin, a cytokine expressed by skeletal muscle cells that is a member of the transforming growth factor (TGF)-β super-family, is usually associated with catabolic effects during skeletal muscle differentiation and growth [71]. Further, myostatin also seems to induce negative effects on bone [71] by several different mechanisms, such as by increasing the expression of sclerostin, dickkopf 1, and nuclear factor kappa β ligand (RANKL) [72], leading thereby to higher osteoclast differentiation [73], culminating in higher bone resorption. Reduced myostatin signaling is associated, not only with skeletal muscle hypertrophy, but also with higher bone density and strength [74], as can be observed in young adult mice treated with a soluble myostatin decoy receptor that displayed an increased muscle mass and bone volume fraction (BV/TV) at the femur and lumbar spine [75]. Regarding BS, myostatin was found to be decreased at six months after RYGB [67] and 18 months after BPD [76], which possibly represents an adaptive response to counteract the skeletal muscle mass progressive decline after surgery. This is suggested by the significant positive correlation between myostatin and fat-free mass loss [76].

Muscle-derived interleukin (IL)-6 is a myokine usually reported as favoring osteoclastogenesis and, thereby, bone resorption [44]. However, when physically stimulated during exercise, skeletal muscles can release IL-6 into the circulation [77], enhancing glucose and fatty acids uptake by myofibers [77]. Importantly, IL-6 induced bone resorption also increases GluOCN levels through an increase in RANKL expression in osteoblasts [44]. Recent evidence suggests that muscle-derived IL-6 can promote nutrient uptake into myofibers in an OCN-dependent manner [78]. Furthermore, mice lacking the IL-6 receptor in osteoblasts present a decrease in exercise capacity that can be corrected by OCN administration [78], highlighting how connected and interdependent is the endocrine crosstalk between bone and skeletal muscle.

When derived from adipose tissue, IL-6 acts mainly as a pro-inflammatory cytokine, and its circulating concentrations are related to whole-body systemic inflammation, such as in obesity [79] and metabolic syndrome [80]. Regarding its response following BS, three months after SG it was possible to observe only changes in IL-6 expression, but not in its circulating concentration [81], whereas at six and 12 months [67] after RYGB, as well as at four years [82] post-surgery (SG, RYGB, BPD or LAGB) it is possible to observe a decrease in adipose tissue-derived IL-6 circulating levels.

Similar to skeletal muscle, adipose tissue has also been described as having a metabolic role through the release of adipokines and hormone-like factors, which also communicate with the skeleton, regulating BM and turnover [83]. Furthermore, adipocyte-secreted molecules can also affect bone quality through the bone marrow by driving the differentiation of mesenchymal stem cells towards the adipocyte lineage at the expense of the bone tissue lineage due to the activation of the master regulator of adipogenesis, the peroxisome proliferator-activated receptor-gamma (PPAR-*ɣ*) [84].

Leptin is an adipocyte-derived hormone that influences the levels of thyroid hormones, growth hormone, cortisol, and sex steroids [85]. Besides, leptin can also regulate bone remodeling indirectly by interacting with the sympathetic nervous system with catabolic effects, as well as directly through hormonal interaction with osteoblasts, which is more related to anabolic effects [85]. Due to this dual interaction, the exact effect of leptin on bone remodeling is not fully understood. Some studies with experimental animal models report that leptin administration can inhibit BM accrual through an increase in sympathetic activity [86, 87], whereas others show that leptin administration results in bone formation [88, 89]. Interestingly, there is also evidence that mice with reduced leptin signaling can present lower BM at the appendicular skeleton, while increases in the trabecular bone at the spine are observed [89, 90], evidencing a caveat in the literature regarding the mechanisms of interaction and effects of leptin in bone. Otherwise, in humans, leptin circulating levels are usually associated with positive effects on bone health [85]. Positive correlations were found between leptin and BMD [91] and a lower risk of fractures [92]. A study carried out with older adults (*n* = 3075) found an inverse correlation between leptin concentration and fracture rates in women, however, this correlation was attenuated when adjusted for age, race, and BMI [92]. Interestingly, a meta-analysis including 70 studies also observed reduced leptin levels post-BS [93]. Therefore, despite some convergent findings, results are still controversial due to confounding factors that also correlate with BMD, such as FM and body weight, further, most of the available evidence is from cross-sectional studies, hindering the possibility of finding a causal relationship between leptin levels and bone health.

Adiponectin is associated with anti-inflammatory effects and is inversely correlated with metabolic syndrome components [94]. In patients with T2DM, serum adiponectin levels were lower than in healthy controls, indicating the inverse correlation between adiponectin and glycemic status [95]. In bone, adiponectin interacts with receptors present in bone cells, especially adiponectin receptor 1 (AdipoR1), inducing the expression of osteogenic genes, such as osteopontin and alkaline phosphatase [96], whereas in mesenchymal stem cells, AdipoR1 knock-down leads to decreases in OCN, osteoprotegerin (OPG), and alkaline phosphatase genes expression [97]. Beyond this, in a bone marrow environment, adiponectin can induce osteoblastogenesis [96, 97], as well as inhibit osteoclast differentiation [98]. In humans with obesity, circulating adiponectin levels are usually decreased, which seems to be related to impaired insulin sensitivity [54] Regarding BS, despite some evidence reporting no differences between adiponectin levels before and after surgery (SG, RYGB, BPD, or LAGB) [82], a recent meta-analysis including 54 studies showed an increase in adiponectin levels after surgery [93], strongly suggesting the direction of this adipokine response to BS.

**Fig. 1:**
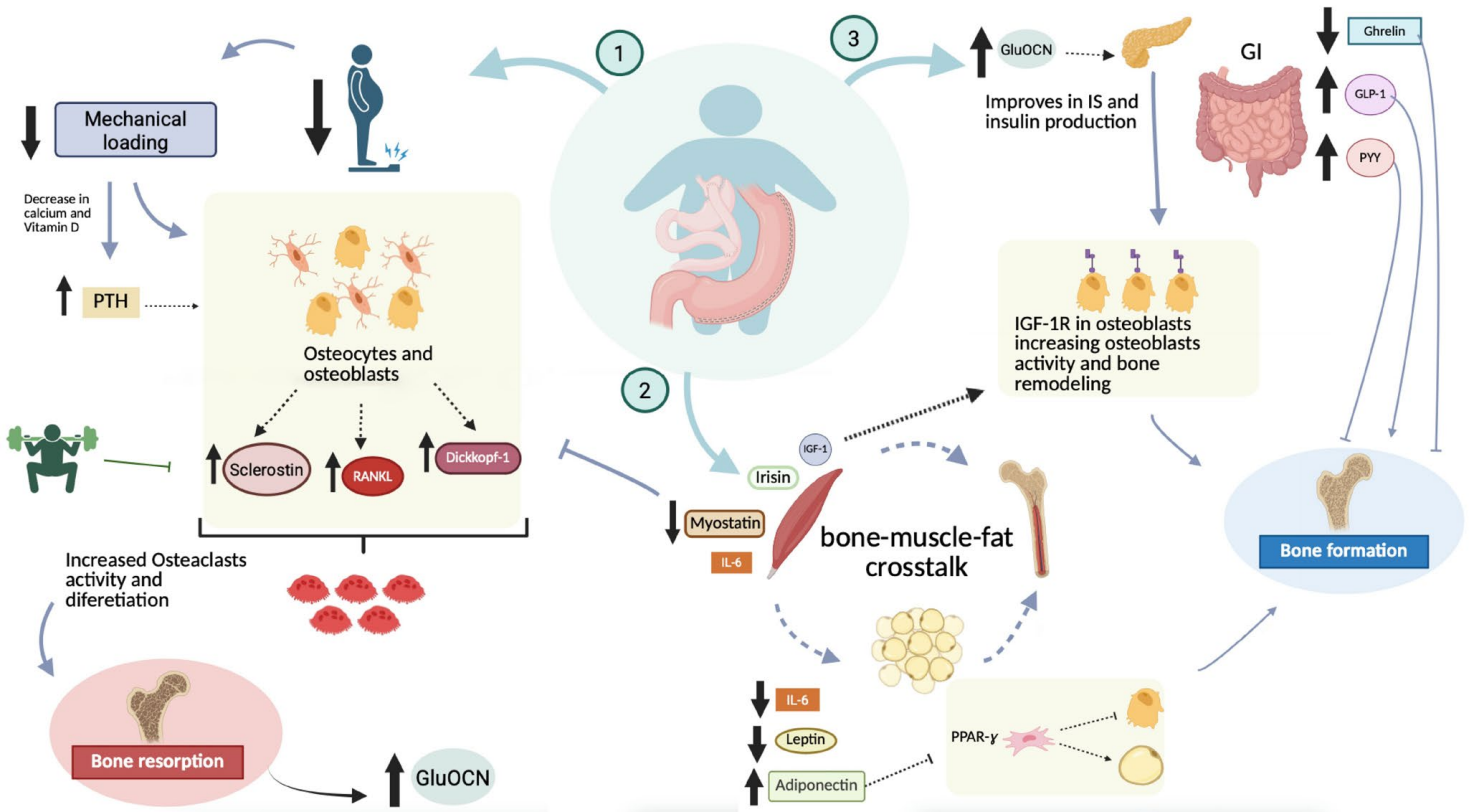
Mechanisms by which BS might affect bone health. (1). WL from BS reduces mechanical loading, leading to higher sclerotin and osteoclastic proteins, increasing bone resorption. PTH rises due to lower vitamin D and calcium, further boosting bone resorption. Increased bone resorption releases GluOCN, influencing overall body functions. Exercise is a viable strategy to lower sclerotin levels; (2). BS affects bone-muscle-fat communication. Myostatin decreases in muscles, countering its negative impact on bones. IGF-1 levels tend to rise post-BS, stimulating bone formation through osteoblasts. IL-6 effects on bones are complex, and dependent on muscle contraction. Leptin decreases, while adiponectin increases after BS, promoting osteogenic differentiation via Adipo-1 receptor; (3). GluOCN released post-BS improves IS, benefiting osteoblast differentiation and bone remodeling. GI feedback changes with higher PYY and GLP-1, and lower ghrelin levels. GLP-1 has an osteogenic effect, while PYY doesn't. Reduced ghrelin post-BS negatively impacts bone quality and density. Abbreviations: weight loss, WL; BS, bariatric surgery; PTH, parathyroid hormone; GluOCN; uncarboxylated osteocalcin; IGF-1, insulin grow factors 1; IL, interleukin; PPAR-y, peroxisome proliferator-activated receptor-gamma; IS, insulin sensitivity; GI, gastrointestinal; PYY; peptide YY; GLP-1, glucagon-like peptides

In an overall view, bone, muscle, and adipose tissue are endocrine organs that are connected by, sometimes physical, but mostly by cytokines crosstalk, leading to many independent interactions and signaling between them. BS, in turn, promotes several changes in endocrine metabolism through several mechanisms that, although not fully elucidated in the literature, lead to increased BM losses. Energy deficiency due to a severe nutritional restriction in the first months following BS seems to be one of the major explanations for the catabolic effects in musculoskeletal structure, as can be observed by how most osteokines and myokines respond to surgery. Otherwise, energy deprivation is the major contributor to weight and FM losses, which seems to positively affect adipokines, inducing a lower systemic inflammation (Fig. 1).

### Crosstalk between Bone and Skeletal Muscle during Exercise

Since exercise promotes beneficial effects on bone remodeling [99], the recognition of the pathways underlying the crosstalk between bone and skeletal muscle during exercise is relevant for refining exercise training (ET) prescriptions for bone preservation. ET is a potential strategy to mitigate bone loss in many populations, including those characterized by uncontrolled bone turnover, such as patients receiving glucocorticoid treatment, and patients with obesity subjected to a restrictive diet [26, 100].

In patients (*N* = 84) who underwent RYGB or SG, Diniz-Sousa et al. investigated whether an 11-month supervised multicomponent exercise program including balance, high-impact, and resistance exercises could induce benefits on bone turnover markers, calciotropic hormones, sclerostin, and BMD. Although they found a relevant exercise treatment effect on BMD, the authors did not find a significant difference in bone biochemical markers [101]. The BABS Study also analyzed 220 patients who underwent RYGB or SG procedures combining calcium, vitamin D, and protein supplementation, along with an ET intervention of two years, including nordic walking, strength perseverance, and equipment training, and they found BMD losses to be partially mitigated in patients with obesity after both surgical procedures [33]. Similarly, preliminary research also assessed bone remodeling markers and BMD before and after supervised weight-bearing and aerobic ET in patients with obesity (*N* = 39) who underwent RYGB. After one year of intervention, they found reduced bone loss in these participants [102]. Moreover, Murai et al. studied women (*N* = 70) with severe obesity who underwent RYGB and who afterwards engaged in a supervised ET program for 6 months. They found that strengthening exercises and aerobic exercise on a treadmill mitigated BS-induced bone loss, probably through pathways involving suppression in bone turnover, as well as sclerostin [103].

Sclerostin, a glycoprotein that inhibits osteoblast differentiation and is a potent inhibitor of osteoblastogenesis [104], is elevated in states of unloading and may regulate alterations in bone metabolism related to WL and exercise [105], thus arising as a candidate in an integrative investigation of molecular pathways regarding bone formation. In this sense, the regulation of sclerostin levels emerges to be a finely tuned pathway by which osteocytes control local and regional osteogenesis in response to changes in mechanical stimulation [105]. Even minor increments in exercise levels can lead to reductions in serum sclerostin [106]. This suppression of sclerostin seems to be a key mechanism mediating the effects of exercise on the skeleton that is postulated as a potential link between mechanical loading and osteoporosis [107]. Available evidence suggests that sclerostin activity inhibition by neutralizing antibodies can promote anabolic effects similar to those induced by mechanical strain [108]. Indeed, romosozumab, a monoclonal neutralizing antibody that binds sclerostin, has been shown to reduce bone resorption, elevate bone formation, and reduce vertebral fracture risk in postmenopausal women [109]. Armamento-Villareal et al. studied older adults with obesity (*N* = 107) who were randomly assigned to either a control, diet-induced WL, ET (aerobic exercise and progressive resistance training), or combined diet-ET group. After 52 weeks of intervention, they found that the elevation in sclerostin concentrations (followed by WL) was prevented by ET, which might partially regulate the negative effects of WL on bone metabolism, thereby evidencing the osteoprotective effect of ET [26] (Fig. 1).

The fact that bone is an endocrine organ that modulates energy metabolism [110] has speeded the idea that bone-derived hormones could be relevant modulators of myokine production and adaptation to exercise. Therefore, bone-derived hormones, such as OCN, as well as myokines (e.g., IL-6), could be related to the feed-forward loop between skeletal muscle and bone that foments adaptation to exercise [44, 111]. In vitro data showed that adding exogenous IL-6 to cultured calvaria osteoblasts elevates the expression of RANKL, a cytokine that facilitates osteoclast differentiation, and reduces OPG, a decoy receptor for RANKL and an inhibitor of bone resorption [112]. Speculatively, these findings suggest that in a living organism, IL-6 may react to exercise by stimulating cells in the osteoblast lineage to increase the production of bioactive OCN and bone resorption. However, at this moment, molecular and genetic studies are still necessary to fully recognize the control of bioactive OCN by IL-6 during exercise. In a mice model in which exercise-induced IL-6 circulating concentrations are reduced (Gprc6aMck−/−), Mera et al. found that, after a single bout of endurance exercise, bone resorption was attenuated. Besides, the elevation in blood biomarkers of bone resorption and in bioactive OCN that is noted in wild-type mice is blunted in this animal model [44].

The feed-forward loop between muscle (via IL-6) and bone (via OCN) foments adaptation to ET by at least three distinct synergistic pathways: (i) OCN elevates catabolism and nutrient uptake in myofibers; (ii) OCN provokes the rise in skeletal muscle expression and secretion of IL-6; and (iii) IL-6 elevates the production of bioactive OCN [113]. It is feasible that IL-6 and OCN have further roles in regulating adaptation to exercise, and this hypothetical model does not exclude this possibility. For a comprehensive discussion of other myokines involved in the pathogenesis of bone diseases, the reader is directed to excellent recent reviews [114, 115] (see Fig. 1).

## Effect of Changes in Gastrointestinal Hormones and Peptides Induced by Bariatric Surgery on Bone Health

Peptide hormones respond as signaling molecules, triggering autonomic reflexes to control energy, appetite, and glucose homeostasis. These peptides are separated into long-term effectors, including insulin, leptin, and adiponectin; and short-term effectors, such as peptide YY (PYY), ghrelin, glucagon-like peptides (GLP)-1 and − 2, and cholecystokinin (CCK) [116]. In addition to the relation between significant body WL and BMD in the first years after RYGB [117], studies have found that a variety of adipocytokines and GI hormones are abnormally produced after BS, which has been suggested to be a relevant predictor of bone loss [118, 119]. In patients with obesity, neural, gut- and adipose-tissue-derived hormone signaling behaves differently [120], presenting, thereby, low fasting concentrations of GLP-1 and PYY and blunted meal-stimulated levels of these anorectic peptides. Therefore, the success of BS approaches to obesity treatment is secondary to changes in GI feedback signaling and elevated secretion of lower gut peptides such as GLP-1 and PYY [121].

There is still scarce evidence on the effect of several GI hormones on bone metabolism, and most of this evidence comes from research performed in vitro and in animal models [122, 123]. For instance, in rodents, GLP-2 administration induced a decrease in bone resorption markers, while GLP-1 triggered an anabolic effect on bone [124, 125]. Using a model of jejuno-intestinal bypass (JIB), le Roux et al. also found increased GLP-1 and PYY in rats submitted to JIB compared with sham-operated rats [126], while PYY has been positively associated with bone resorption in mice [122]. Besides, there is some evidence that ghrelin seems to increase the proliferation and differentiation of osteoblastic cells [127].

In humans, Carrasco et al. investigated changes in BMD after one year of BS, including both LAGB and RYGB, and found a relevant decrease in BMD only after RYGB, being the reduction in ghrelin being the major variable correlated with bone losses [117]. Guerrero-Pérez et al. also showed that fasting ghrelin after RYGB in patients with T2DM had an inverse correlation with bone mineral content at the lumbar spine. The authors suggest that the increase in ghrelin levels after BS, as a compensatory reaction to the negative energy balance at the time of maximal bone loss, might explain this negative relationship [119].

Compared to ghrelin levels, more solid results have been found for the effects of BS in humans following different BS techniques on plasma concentrations of PYY and GLP-1. In this sense, following RYGB, but not gastric banding, plasma concentrations of PYY and GLP-1 are notably elevated [126, 128]. In fact, the meal-stimulated GLP-1 and PYY secretion are relevantly increased as early as two days after RYGB surgery and continue to increase up until six weeks of follow-up [129]. In a pilot study, Rhee et al. investigated the impact of RYGB on the changes in density and gene expression of small-intestine enteroendocrine cells in patients with obesity and with (*N* = 12) or without (*N* = 11) T2DM. About 10 months after the BS, they found numerous changes in the distribution of enteroendocrine cells and their expression of hormonal genes, including elevated density of PYY, CCK, GLP-1, glucose-dependent insulinotropic polypeptide (GIP), and prohormone convertase 2-positive cells, and decreased gene expression of secretin, ghrelin, and GIP [130].

After RYGB, because the gastric lumen is reduced, only a portion of the usual gastric volume can be accommodated, and antral trituration and pyloric control of emptying are absent. As a consequence, RYGB accelerates gastric emptying [131], which, in turn, often results in nausea, bloating, and dumping syndrome in these patients [132]. Such quick raises in small intestine nutrient content probably contribute to the elevated meal-related secretion of insulin, GLP-1, CCK, and PYY [3–36] after RYGB. This is demonstrated, for example, by using the infusion of glucose at a high physiological rate (4 kcal/min) into the duodenum of healthy subjects. Nguyen et al. found that the Roux limb of RYGB patients induced comparable raises in GLP-1, whereas research in RYGB patients using oral glucose loads (200 kcal/150 ml) presented larger GLP-1 responses [133] (see Fig. 1).

## Future Perspectives

Results suggest a tendency for increased risk of bone fracture after RYGB [134], as RYGB was the most commonly performed BS worldwide until it was very recently overtaken by the SG. This increased risk of bone fracture is in line with evidence from studies showing a sharp increase in bone resorption [135] and a decrease in BM at the axial skeleton after RYGB [17]. Whether the higher risk of fracture is only due to increased bone fragility or also to an elevated risk of falls is still an open question. This may be connected to the fact that RYGB is recognized as the most malabsorptive surgical procedure. Consequently, we cannot dismiss the possibility of increased mineral deficiencies that are important for bone health following this surgical technique. The superior WL after RYGB is another fact that must be considered regarding bone remodeling markers, since mechanical unloading upregulates sclerostin expression, thereby stimulating osteoclastogenesis [28]. Furthermore, as weight decreases, not only FM might be reduced but also LM, further impairing bone health [136].

The various comorbidities frequently present in patients with severe obesity, such as the presence of T2DM, make it hard to find a clear pattern in some cytokine responses to BS. Besides, further studies are also needed to determine the exact role of insulin in bone metabolism in these patients [137, 138]. Above all, it remains largely unclear how BS induces changes that modify the secretion of various hormones, many of which have opposing effects and interfere with bone metabolism.

Body composition changes induced by BS are hypothesized to induce alterations in the concentration of sclerostin and adipokines [139, 140], which could regulate the expression of the RANKL and OPG [141, 142], with bone repercussions proportional to WL [143, 144]. Since the pathophysiological pathways involved in these changes are not fully explained, research on the relationship between bone remodeling and these proteins after BS is needed. Besides, molecular and genetic studies are still required to fully understand the regulation of bioactive OCN by IL-6 during exercise.

Exercise for bone health should be primarily different from that for metabolic and cardiovascular health [145, 146]. Although the general literature has suggested that weight-bearing with intensive ET should be more successful in stimulating bone formation [147], the ideal type of ET to counteract bone loss after BS remains unknown. It is also reasonable to consider that additional molecules produced by muscle or bone cells could play a role in the communication between bone and skeletal muscle during exercise. Future studies are necessary to thoroughly examine these hypotheses in humans and confirm results from animal models. Furthermore, it’s important to note that certain benefits of ET on the skeleton may diminish after exercise cessation. Therefore, it is important to investigate whether, and to what extent, ET can prevent fractures in the long term. Furthermore, it is also necessary to investigate how the organs are targeted by bone-derived biochemical signals to further elucidate how the skeleton might preserve organismal homeostasis.

## Final Considerations

Bone, muscle, and adipose tissue are endocrine organs that are connected by cytokines crosstalk, and this crosstalk is performed by myokines, osteokines, as well as growth-like factors, which affect, afterward, the musculoskeletal metabolism. For instance, IL-6-induced bone resorption increases GluOCN levels through an increase in RANKL expression in osteoblasts, which highlights how connected and interdependent the endocrine crosstalk between bone and skeletal muscle. Moreover, GluOCN can also regulate insulin secretion and sensitivity in peripheral tissues, evidencing a bone-pancreas endocrine loop.

The marked BS-derived WL may lead to a substantial decrease in mechanical stimulation on bone tissue and a consequent increase in the serum level of sclerostin secreted by osteocytes, thereby decreasing bone formation, even though some results contradict this hypothesis. Besides, patients undergoing BS often exhibit high levels of PTH and secondary hyperparathyroidism due to calcium and vitamin D deficiency. The decrease in calcium and vitamin D absorption after BS leads to a compensatory hormonal response mediated by PTH to keep calcaemia within physiological limits by increasing calcium mobilization from its largest reservoir, which is bone tissue, gradually favoring BM loss.

Myostatin seems to induce negative effects on bone by increasing the expression of sclerostin, dickkopf 1, and RANKL, thereby leading to higher osteoclast differentiation and higher bone resorption. In addition, irisin is also involved in skeletal muscle-bone crosstalk, which is correlated with bone health through an increase in osteoblastogenesis and bone formation, but the results related to BS patients are still controversial. In humans, leptin and adiponectin levels are usually associated with positive effects on bone health (e.g., positive correlations between leptin and BMD, and a lower risk of fractures), however, their exact effect on bone remodeling is not fully understood.

OCN, as well as myokines, could be related to a feed-forward loop between skeletal muscle and bone that foments adaptation to exercise. Moreover, even minor increments in exercise levels can lead to reductions in serum sclerostin. This suppression of sclerostin seems to be a potential link to prevent the increased risk of fractures and osteoporosis through the mechanical loading approach.

The success of BS approaches to obesity treatment is secondary to changes in GI feedback signaling and elevated secretion of lower gut peptides such as GLP-1 and PYY. However, there is scarce evidence on the effect of several GI hormones on bone metabolism, and most of this evidence comes from research performed in vitro and animal models.

## Data Availability

No datasets were generated or analysed during the current study.
